# Identification of novel cattle (Bos taurus) genes and biological insights of their function in pre-implantation embryo development

**DOI:** 10.1186/s12864-024-10685-5

**Published:** 2024-08-09

**Authors:** Gustavo P. Schettini, Michael Morozyuk, Fernando H. Biase

**Affiliations:** School of Animal Sciences, Virginia Polytechnic Institute and State University, Blacksburg, VA USA

**Keywords:** Gene annotation, Embryos, Transcriptome

## Abstract

**Background:**

Appropriate regulation of genes expressed in oocytes and embryos is essential for acquisition of developmental competence in mammals. Here, we hypothesized that several genes expressed in oocytes and pre-implantation embryos remain unknown. Our goal was to reconstruct the transcriptome of oocytes (germinal vesicle and metaphase II) and pre-implantation cattle embryos (blastocysts) using short-read and long-read sequences to identify putative new genes.

**Results:**

We identified 274,342 transcript sequences and 3,033 of those loci do not match a gene present in official annotations and thus are potential new genes. Notably, 63.67% (1,931/3,033) of potential novel genes exhibited coding potential. Also noteworthy, 97.92% of the putative novel genes overlapped annotation with transposable elements. Comparative analysis of transcript abundance identified that 1,840 novel genes (recently added to the annotation) or potential new genes were differentially expressed between developmental stages (FDR < 0.01). We also determined that 522 novel or potential new genes (448 and 34, respectively) were upregulated at eight-cell embryos compared to oocytes (FDR < 0.01). In eight-cell embryos, 102 novel or putative new genes were co-expressed (|r|> 0.85, *P* < 1 × 10^–8^) with several genes annotated with gene ontology biological processes related to pluripotency maintenance and embryo development. CRISPR-Cas9 genome editing confirmed that the disruption of one of the novel genes highly expressed in eight-cell embryos reduced blastocyst development (ENSBTAG00000068261, *P* = 1.55 × 10^–7^).

**Conclusions:**

Our results revealed several putative new genes that need careful annotation. Many of the putative new genes have dynamic regulation during pre-implantation development and are important components of gene regulatory networks involved in pluripotency and blastocyst formation.

**Supplementary Information:**

The online version contains supplementary material available at 10.1186/s12864-024-10685-5.

## Background

In mammals, during folliculogenesis, a subset of oocytes leave their quiescent state and progress through oogenesis. Specially in the growth phase [1–3], oocytes accumulate maternal transcripts [4, 5] produced from over 15 thousand genes and store thousands of proteins [6, 7]. The coordinated regulation of transcriptional activation or repression is critical for a successful embryo development. Upon fertilization, the maternal storage of RNAs and proteins are sufficient for the embryo to undergo a few cleavages independent of embryo genomic activation [8, 9]. Further development, however, depend on the transcription of embryonic genes that starts at 2-cell stage and expands significantly when the embryo reaches the 8-cell stage [10]. A dynamic regulation of gene activation and repression continues through compaction, blastulation [11, 12] and further cellular differentiation.

Oocytes and pre-implantation embryos express thousands of genes [5, 11, 13–17], among which are protein-coding genes, long non-coding genes, small RNAs and other biotypes. Understanding the genetic mechanisms underlying oocyte and embryo development is crucial for unveiling the causes of imbalance that lead to developmental arrest. The assessment of transcriptome profiles using high throughput sequencing has been fundamental in shedding light on gene expression differences between oocyte and embryo stages in cattle [11, 18, 19]. Data from high throughput sequencing have also been used to enhance functional annotation by discovering novel mRNA isoforms [20, 21] and other potential classes of RNA molecules in oocytes and embryos at different developmental stages [22–25]. However, most of the research conducted to date have used high throughput sequencing of short-reads.

While high throughput sequencing of short-reads provide accurate sequences at massive abundance, their ability to detect long transcripts is limited due to the maximum read length of 150 nucleotides [26]. In contrast, long-read sequencing technologies such as Pacific Biosciences and Oxford Nanopore Technology (ONT) have demonstrated the capability to generate sequences greater than 10kb and detect full-length transcripts [27, 28]. By exclusively utilizing ONT long-reads, Halstead et al. [29] identified several unknown transcripts in 32 tissues from Hereford cattle breed, including tissue-specific isoforms. This discovery unveiled potential unannotated mRNA isoforms and non-coding RNA classes that are missing from the current official cattle annotation and may play key roles in biological processes.

Despite the ability of long-read sequencing technologies such as ONT to detect full-length transcripts, efforts to improve flow cells, chemistry, and basecalling algorithms have increased the accuracy, since these technologies have been prone to higher error rates compared to short-read sequencing methods [26, 30]. On the other hand, the combination of short-read and long-read sequencing technologies has shown significant advantages in the genome assembly in cattle [31–34] and other livestock species [35–38] since this hybrid approach has facilitated the discovery of new transcripts, loci, identification of structural variants, gap closure, and improved sequence accuracy. Also, it has become the preferred method for reconstructing genomes and transcriptomes in various organisms, as it enables the generation of more accurate and longer contig and scaffold sequences by leveraging both long-read and short-read sequencing data [30, 39].

Despite the benefits of combining long and short sequences for transcriptome reconstruction, limited studies have assessed the transcriptome profile of cattle samples using this hybrid approach. A recent study [40] has identified several thousand new genes in the cattle genome, however oocytes and pre-implantation embryos were not represented in their samples. Here, we hypothesized that there are several genes expressed in oocytes and pre-implantation embryos that are not yet annotated and many of those genes are functionally important for embryo development. Therefore, we aimed to (a) de novo reconstruct the transcriptome of oocytes and pre-implantation embryos using short- and long-sequences, and (b) evaluate the potential role of those new genes in pre-implantation embryos. A comprehensive analysis of novel genes and putative new genes (not yet annotated) provides new insights into the complex gene regulatory networks at the time of embryo genomic activation and blastocyst formation.

## Methods

**Fig. 1:**
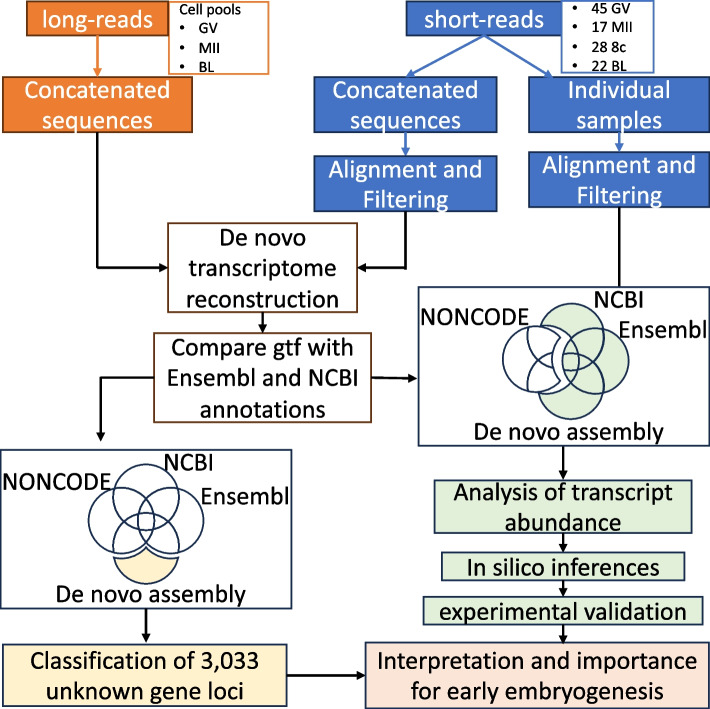
Overview of bioinformatic steps for transcriptome reconstruction and identification of unknown gene loci, followed by categorization and functional categorization. GV: oocyte in the germinal vesicle stage; MII: oocyte in the metaphase II stage; 8c: eight cell stage embryos; BL: blastocysts

No live animal was handled for this work, thus approval by the Institutional Animal Care and Use Committee was not necessary. An overview of the work conducted is presented in Fig. 1.

### Sample collection

All procedures for oocyte maturation, embryo culture and media composition were described in detail elsewhere [41–43]. *Bos taurus* ovaries were collected from a commercial slaughterhouse and transported to the laboratory in a 0.9% saline solution and antibiotic and antimycotic solution (1x, ThermoFisher Scientific, Waltham, MA, USA). First, ovarian follicles were slashed, and the cumulus-oocyte complexes (COCs) were retrieved from the follicular fluid into oocyte collection media (OCM). Next, we assessed the morphological properties of the COCs under a stereoscope and only COCs presenting homogeneous ooplasm and more than three layers of compact cumulus cells were selected for sample collection or in vitro maturation (IVM) at the density of ten COCs in 50μl of IVM medium in a humidified incubator at 5% CO_2_ and 38.5 °C for 22–24 h.

For in vitro fertilization (IVF), we washed in vitro matured COCs in 4-(2-hydroxyethyl)-1- piperazineethanesulfonic acid (HEPES) buffered synthetic oviductal fluid (HEPES-SOF), followed by two washes in fertilization medium (SOF-Fert). The IVF was set up in SOF-Fert with the addition of sperm (1,000,000 sperm/ml) and incubation for 16–18 h in a humidified incubator at 5% CO_2_ and 38.5 °C. In vitro culture (IVC) of putative zygotes was conducted by removal of cumulus cells adhered to the zona pellucida in HEPES-SOF, which was followed by three washes in SOF culture media (SOF-BE). Then, we placed 25–30 presumed zygotes, in micro drops (50µl) of culture media covered by mineral oil and in a humidified incubator at 38.5 °C under 5% CO_2_, 5% O_2_, and 90% N_2_.

We collected oocytes at the germinal vesicle stage and metaphase II stage by washing the COCs in Trypsin (TrypLE™ Express, gibco, Grand Island, NY) twice followed by a 10-min incubation. We removed the remaining cumulus cells adhered to the zona pelucida by repeated pipetting. Then, we washed the oocytes in phosphate buffered saline solution (PBS, ThermoFisher Scientific, Fair Lawn, NJ). Embryos were collected at either 60 h post culture (at 8-cell stage) or 164 h post culture (at blastocyst stage) and washed twice in PBS. Oocytes were collected either individually in 1µL of PBS or in groups of 50 in 5 µL of PBS. Embryos were collected either individually in 1µL of PBS or in groups of 25 embryos in 5 µL of PBS. All samples were preserved at -80 °C until RNA extraction.

### RNA extraction and Hight-throughput sequencing

We extracted total RNA from all samples using TRIzol reagent [44] with Phasemaker Tubes using the procedures described elsewhere [45], followed by storage at -80 °C until further processing.

To conduct short read high throughput sequencing, we prepared libraries of single oocytes or single embryos (45 individual oocytes at the GV stage, 17 oocytes at the MII stage, 28 8-cell embryos, and 22 blastocysts) using a procedure described elsewhere [45, 46]. Briefly, RNA pellets were resuspended into a solution containing oligo-dTVN oligonucleotide (1mM) (Additional file 1) and heated in a thermocycler at 72 °C for three minutes. Reverse transcription was carried out by adding 5µl of a solution containing 200 U/µl Maxima H Minus Reverse Transcriptase, 1 × Maxima RT Buffer, 7.5% PEG 8000, 10 mM dNTPs, and 2 µM of a template-switching oligo (Additional file 1) and to the RNA oligonucleotide mix for 1h 30min at 42°C. Next, cDNA was purified by AMPure XP beads.

Promptly after cDNA purification procedures, an amplification mix containing 1.25U Terra polymerase, 1 × Terra direct buffer, and 0.1µM of cDNA amplification primer (Additional file 1) was added to each sample tube. We used purified cDNA for amplification by polymerase chain reaction (PCR) under the following conditions: 98 °C for 15 min, 68 °C for 5 min, and 72 °C for 10 min, and presumed at 8 °C after the last cycle. The amplification products were cleaned using AMPure XP magnetic beads, quantified with a Qubit 4.0 fluorometer, and quality-assessed by 2100 Bioanalyzer and the Agilent High Sensitivity DNA kit.

According to the manufacturer’s instructions, we used 1ng of cDNA as a template for library preparation with the Nextera DNA Flex Library Prep kit. Followed by PCR amplification (13 cycles) and a purification step using AMPure XP beads. We quantified the libraries using a Qubit 4 fluorometer and assessed their quality using the Agilent High Sensitivity DNA kit in a 2100 Bioanalyzer. Libraries were sequenced at Vanderbilt Technologies for Advanced Genomics at Vanderbilt University – VANTAGE to produce 150 bp pair-end reads in a HiSeq 2500 or NovaSeq 6000 Illumina sequencer (Illumina, Inc. San Diego, CA).

To proceed with high throughput sequencing of long reads, collected samples of each development stage (GV, MII, and BL) in groups of 50 specimens, and extracted RNAs from each pool of samples. Next, we amplified the material with TeloPrime Full-Length cDNA Amplification Kit V2 (Lexogen, Vienna, Austria), following the manufacturer’s procedures and 20 PCR cycles. Next, the libraries were prepared with the Native Barcoding kit 24 V14 (Oxford Nanopore Technologies, Oxford, England). We sequenced the libraries using the MinION Mk1C sequencer with R9.4.1 (GV oocytes) and R10.4.1 (MII oocytes, and BL) flow cells (ONT Ltd., Oxford, United Kingdom).

### Pre-processing RNA-seq sequences

We trimmed the adaptors from short-reads sequences produced from 45 GV, 17 MII, 28 8c, and 22 BL samples with Trimmomatic v.0.39 [47] and aligned to the cattle genome (ARS-UCD 1.2) [31] downloaded from the Ensembl database [48], using HISAT2 v2.1.0 aligner [49]. Next, we used SAMtools v1.10 [50] and biobambam2 v2.0.95 [51] to remove unmapped reads, secondary alignments, PCR duplicates, and duplicated sequences. Later, we converted those filtered alignment files (.bam) to fastq files using the *bomtofastq* command built on the biobambam2 v2.0.95 [51]. Then, aiming to increase specificity, we concatenated and pre-processed all sequences on BBTools (Bushnell B.; https://www.sourceforge.net/projects/bbmap/) to maintain regions with coverage between 10 × and 30 × coverage.

We conducted base calling of ONT long-reads using Guppy v.6.4.2 [52] with the super accuracy algorithm (*dna_r10.4.1_e8.2_260bps_sup.cfg*), set to remove low-quality reads (< Q10). The resulting reads used in the de novo assembly had an average quality greater than Q24.

### Hybrid de novo transcriptome assembly

We conducted transcriptome assembly based on short and long sequences using the hybrid de novo assembler rnaSPAdes v3.14.1 [53]. The de novo assembly by RNAspades relies mainly on the SR sequences and only uses the LR sequences as support to close gaps between SR contigs [53].

### Unknown or novel gene loci identification

First, we aligned the sequences obtained from the de novo assembly to the *Bos taurus* genome (ARS-UCD 1.2) using the GMAP aligner (version 2021–12-17) [54] to produce a preliminary annotation (.gff3) and alignment (.sam) using the following parameters to improve accuracy and report only the best sequence paths (*–microexon-spliceprob* = *1 –nofails –quality-protocol* = *illumina –suboptimal-score* = *0.99 –min-identity* = *0.90 –npaths* = *1*). Sequence alignments with more than ten mismatches were filtered out.

Second, we compared our assembly to the Ensembl annotation file (ARS-UCD 1.3.111) with gffcompare v0.12.6 [55] to identify loci not yet present in the Ensembl annotation. Only transcripts classified with the flag “-u” (unknown) were retained in our annotation file. The fasta sequences from transcripts listed as unknown relative to the Ensembl annotation were aligned to the NCBI [56] genome (ARS-UCD 1.2) and mapped to the RefSeq annotation (GCF_002263795.3-RS_2023_09). Only transcripts classified with the flag “-u” (unknown) were retained in our annotation file.

Third, we compared the transcripts identified as not present in both Ensembl and NCBI databases to the lncRNA database (NONCODEV5) [57]. Lastly, we filtered out any unknown transcript located within five kilobases to the boundaries of known genes, containing only one exon and those with less than 300nt length from our annotation file by gffread v0.12.8 [55] and GenomicRanges R-package [58].

We used the annotation file (unknown loci) to count short-reads by FeatureCounts [59]. We retained loci with transcript abundance greater than two counts per million (CPM) in five or more samples. Subsequently, we used gffread [55] and bedtools [60] to reduce the redundancy of our assembly by merging transcripts into unknown gene loci allowing a maximum gap of 200 nucleotides.

### Classification of Unknown loci

In order to classify the unknown gene loci identified in our study, we assessed the coding potential based on high similarity with non-redundant protein (nr) sequences deposited in NCBI database v5 (ftp.ncbi.nlm.nih.gov/blast/db/v5/FASTA—updated on 07/Feb/2024) via DIAMOND v.2.0.11.149 [61]. We used database for potential protein family members located in different regions in cattle (*Bos taurus; Bos indicus; Bos taurus x Bos indicus*), followed by orthologous proteins in the mammalian class, prioritizing human (*Homo sapiens*) and mouse (*Mus musculus*). First, we carried out a local alignment via DIAMOND using parameters to increase specificity, such as an E-score threshold of 1 × 10^–6^, > 90% of subject coverage, and a percentage of identity greater than 90% to report a potential hit for cattle. Second, the remaining loci with no match went through a local alignment to identify potential orthologs in mammalian organisms. The parameters were similar, except for the percentage of identity threshold, which was set at > 70%. Unknown gene loci that remained without classification were assessed by a neural network classification model, RNAsamba [62], and classified for coding potential on open read frame (ORFs) and untranslated region (UTRs) features. Lastly, we mapped the unknown loci to known transposable elements (TE) by coordinates overlap conducted by GenomicRanges [58]. TE coordinates were obtained from RepeatMasker [63] and retrieved from the University of California Santa Cruz (UCSC) database [64, 65].

### Unknown/novel gene loci abundance and differential expression

We determined loci transcript abundance by counting short-reads mapped to the gtf files obtained from Ensembl (ARS-UCD 1.3.111) and NCBI (GCF_002263795.3-RS_2023_09) and our gtf file with unknown loci via FeatureCounts [59]. Subsequently, we combined the gene raw count matrices and removed NCBI genes with mapped identifiers in the Ensembl annotation.

We normalized libraries using the trimmed mean of M values (TMM) [66], followed by a count per million (CPM) using the edgeR package [67]. Loci with less than five CPM in at least 15 samples were filtered out. Then, we conducted the differential gene expression analyses using EdgeR [67] and DESeq2 [68] R-packages, and loci classified as statistically differentially expressed (DE) in both algorithms if |LogFC|> 1 and FDR < 0.01.

### In silico functional characterization novel or unknown loci

Focusing on pre-implantation embryos, we performed a co-expression analysis on novel genes/potential novel gene loci used for differential expression analysis. The same normalized matrices obtained previously were transformed using an inverse hyperbolic sine [69], followed by Pearson correlation coefficient calculation between the novel genes/potential novel gene loci and Ensembl known genes using WGCNA R-package function *corAndPvalue* [70]. We retained absolute correlation values ≥ 0.85 with a *P* ≤ 0.00005. Further, we conducted a functional enrichment analysis using GOseq R-package [71] on the genes highly co-expressed, and only biological processes (BP) with more than four genes and FDR ≤ 0.01 using the Holm–Bonferroni method considered significantly enriched. Additionally, we functionally characterize the novel genes/potential novel gene loci based on co-expressed annotated gene functional information retrieved from biomaRt [72]. Biological processes containing > 55 co-expressed genes and in at least 50 novel genes/potential novel gene loci correlations were maintained. Finally, co-expression networks were generated by Cytoscape (v.3.10.1) [73].

### Gene editing using CRISPR-CAS9

We designed gRNAs (Additional file 1) to target exon 2 of the novel gene locus (ENSBTAG00000068261/LOC132342749) located on chr18:48,758,182–48,764,129 using the CRISPOR [74]. All gRNAs were purchased as a single RNA molecule (sgRNA) comprising crRNA and transacting crRNA (tracerRNA) from IDT (Integrated DNA Technologies, Research Triangle Park, NC, USA), as well as CRISPR-Cas9D10A nickase V3.

We mixed CRISPR-Cas9D10A and sgRNAs for the formation of ribonucleoproteins in OptiMEM reduced serum medium (Thermo Fisher Scientific, Grand Island, NY) at room temperature for at least 1h before electroporation. The concentrations in the solution for the formation of RNPs were 800ng/µl Cas9D10A and 800ng/µl of each sgRNA. We electroporated the presumptive zygotes (PZ) following the procedures detailed elsewhere [75, 76]. We removed the cumulus cells from the PZs and electroporated them in Opti-MEM media containing RNPs at the concentration of 400ng/µl Cas9D10A and 400ng/µl of each sgRNA. The electroporation parameters were as follows: six poring pulses of 15V, with 10% decay, for 2ms with a 50ms interval, immediately followed by 5 transfer pulses of 3V, 40% decay, for 50ms with a 50ms interval, alternating the polarity. We conducted two electroporation sessions, the first at 14 h post fertilization (hpf) and the second at 20 hpf. After the second electroporation, PZs were placed in culture media and incubated as indicated above.

We recorded the number of embryos that cleaved at ~ 45hpf and blastocysts at ~ 168hpf and ~ 190 hpf. For statistical analysis we considered culture drops as biological replicates. We analyzed count data (success of blastocyst development or developmental arrest) using a general linear model with a binomial family, which results in logistic regression analysis [77], using the “glmer” function from the R package “lme4” [78]. We used the number of blastocysts and the number of putative zygotes that failed to develop into blastocysts as the dependent variable. Group (Cas9 + targeting gRNAs or Cas9 + scramble gRNAs) was a fixed effect and replicate was a random variable. The Wald statistical test [79] was conducted with the function “Anova” from the R package “car” [80]. Finally, we carried out a pairwise comparison using the odds ratio and two-proportion z-test employing the “emmeans” function of the R package “emmeans”. The null hypothesis assumed that the odds ratio of the proportion ($p$) of two groups was not different from 1 (${H}_{0}: {p}_{1}{/p}_{2}=1$). We inferred significance when adjusted *P* value < 0.05.

We executed the experiment in triplicate subjecting 182 and 161 presumptive zygotes to editing procedures with targeting gRNAs and sramble gRNAs, respectively. To access the edits produced, at ~ 190 hpf, we also collected all embryos that arrested their development at the morula stage. We removed their zona pellucida by a treatment with EmbryoMax Acidic Tyrode’s Solution (Millipore Sigma, Danvers, MA) and exposed their DNA by adding four µL of QuickExtract DNA Extraction Solution (Biosearch Laboratories, USA), and incubating the solution at 65 °C for 15 min followed by 2 min at 98 °C and hold at 4 °C.

Then, we conducted PCR reactions using the oligonucleotides described on Supplementary Additional file 1. The oligonucleotides were designed using NCBI’s Primer-BLAST [81] and certified their specificity using the University of California Santa Cruz’s BLAST-Like Alignment Tool available in the Genome Browser [64, 82]. The PCR reaction mix consisted of 1.25 PrimeSTAR GXL DNA Polymerase (Takara Bio USA, San Jose, CA), 1 × Buffer, 200 μM dNTPs, and forward and reverse oligonucleotides (IDT, Coralville, IA, USA) at 0.2 μM each, in a final volume of 50 μL. The cycling conditions for this reaction were: 98 °C for 1 min, followed by 30 cycles of 98 °C for 15 s, 62 °C for 15 s, and 68 °C for 4 min, followed by a final extension of 4 min at 72 °C. We confirmed the amplification by assaying 5 µL of each amplicon by electrophoresis on a 1.5% Agarose gel before staining with Diamond Nucleic Acid Dye and imaging. Finally, we prepared libraries for amplicon sequencing with the Native Barcoding Kit 24 V14 (Oxford Nanopore Technologies, Lexington, MA, USA), following the manufacturer’s instructions. We sequenced the libraries in a MinION flowcell (R10.4.1) using a MinION Mk1C (Oxford Nanopore Technologies, Lexington, MA, USA) following the manufacturer’s recommendations. We carried out super accuracy base calling with Guppy v6.4.2 [52], followed by quality filtering using Fitlong (https://github.com/rrwick/Filtlong) to remove short sequences (< 500nt). Then, filtered reads were mapped to the cattle reference genome from Ensembl (ARS-UCD1.2) using minimap2 v2.27 [83, 84] and sequences with < 500nt aligned with the reference genome were removed by SAMtools [50], as well as secondary alignments.

## Results

### Overview of the sequencing data and hybrid de novo transcriptome assembly

We generated approximately 1.8 billion, 722.49 million, 1.9 billion and 1.05 billion pairs of raw short-reads from 45 oocytes at the germinal vesicle stage (GV), 17 oocytes at the metaphase II stage (MII), 28 embryos at the eight-cell (8c) stage, and 22 embryos at the blastocyst (BL) stage, respectively (Additional file 2). We also produced 23.76 million base-called long-reads (6.03 – GV, 6.01 – MII, and 11.72 – BL) at an average length of 1,417 nucleotides long (minimum: 20; maximum: 91,586).

A flow chart with the schematics of the study is depicted in Fig. 1. For transcriptome assembly, we grouped all short-read sequences for alignment, quality filtering, and coverage normalization (10–30 × range). These processes resulted in 41.66 million short pair-end reads used as input along 23.76 million long sequences into RNAspades assembler [53]. The resulting transcriptome assembly generated 277,818 sequences with a mean length of 2,462 nucleotides (min.: 77; max.: 93,634) (Additional file 3). After aligning to the genome with GMAP [54] we obtained the coordinates for 274,342 transcripts (Table 1).

**Table 1:** Summary of de novo transcriptome reconstruction and mapping to annotation

	Total number of genomic regions	Novel genes
De novo assembly	274,342	-
Ensembl	202,945	7,990
NCBI/RefSeq	10,117	1,062
lncRNA (NONCODEv5)	882	-
Not yet annotated-current study	22,305	3,033

### Novel gene loci identified in oocyte and pre-implantation embryos

After comparisons with reference annotation information from Ensembl (ARS-UCD1.3.111), NCBI (ARS-UCD.2.0.GCF_002263795.3-RS_2023_09), and lncRNA NONCODEv5 databases (Fig. 1), we identified 22,305 transcripts that did not overlap coordinates with the annotations assessed. Those transcripts were reduced to 3,033 loci (see methods for details) that have not yet been annotated, which we referred to as potential novel genes (Table 1).

Based on homology with sequences present in the NCBI database, we determined that 63.67% (1,931/3,033) of the putative novel genes have coding potential, while there was an indication that the remaining 36.33% putative novel genes are transcribed as long non-coding RNAs (Table 2). The majority (97.92%, 1,926 coding; 1,044 noncoding) of the putative novel genes had overlapping coordinates with at least one transposable element (Table 2). There is a greater (99.74%) proportion of potential new genes with coding potential associated with transposable elements relative to the annotated novel genes (93.12%, Pearson’s chi-squared test statistic = 124.06, *P* < 2.2 × 10^–16^, 2-sample test for equality of proportions [85]). This distinct pattern of colocalization with a transposable element is less prominent for non-coding potential new genes (94.74%) relative to annotated new genes (93.12%, Pearson’s chi-squared test statistic = 3.68, *P* = 0.03, 2-sample test for equality of proportions [85]).

**Table 2:** Summary of classification of annotated novel genes and potential novel genes

		Classification	Associated with TE	Not associated with TE	Total
Annotated	Novel genes	Coding	2,786	206	2,992
		Noncoding	5,643	417	6,060
Not annotated	DIAMOND	Coding	1,886	1	1,887
	RNAsamba	Coding	40	4	44
	RNAsamba	Noncoding	1,044	58	1,102

### Expression of novel and putative novel loci during pre-implantation development

**Fig. 2:**
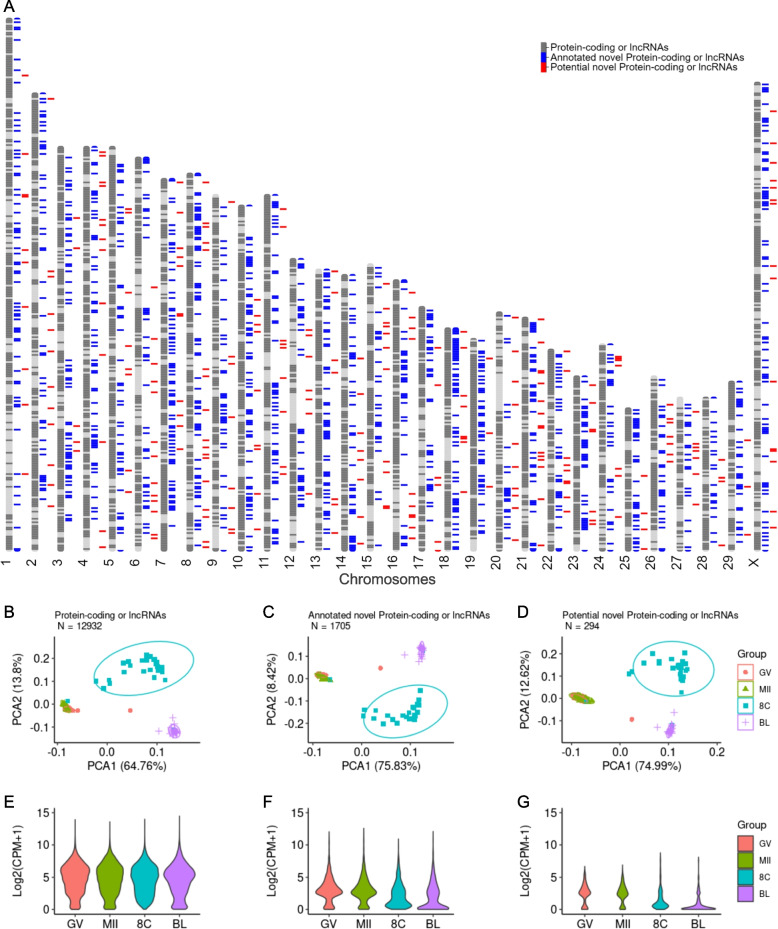
Overview of the transcriptome (protein-coding or long-noncoding genes) of single oocytes or pre-implantation embryos. **A** Distribution of the novel (blue) and potential new genes (red) across the cattle genome. Principal component analysis of annotated (**B**), annotated as novel (**C**) and putative novel genes (**D**). Distribution of the average transcript abundance of annotated (**E**), annotated as novel (**F**) and putative novel genes (**G**)

After filtering lowly expressed genes (< 5 counts per million, CPM and < 15 samples), we retained 12,932 annotated protein-coding or lncRNA genes, 1705 annotated novel protein-coding or lncRNA genes and 294 potential new genes (out of 3,033 shown on Table 1), for which we estimated robust relative transcript abundance. We noted that the potential new genes were distributed across all chromosomes (Fig. 2A). All three gene subsets produced similar global separation of samples, including a similar pattern of dispersion of samples, with the broadest dispersion observed among embryos collected at the 8-cell stage (Fig. 2B-D). From a global perspective, novel annotated and putative new genes had similar patterns of expression, with an overall less transcript abundance across oocytes or embryos, relative to the annotated genes (Fig. 2E-G).

**Fig. 3:**
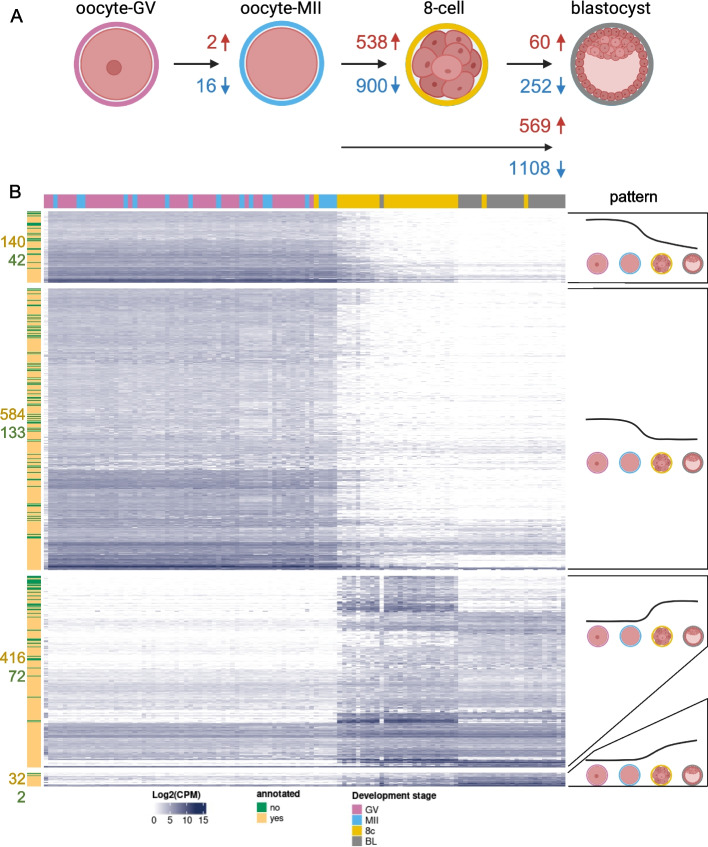
Differential transcript abundance of 1999 novel or putative novel loci during pre-implantation stages. **A** Summary of the number of loci with differential transcript abundance between two stages (|Log2(FC)|> 1 and FDR < 0.01). **B** Patterns of differential transcript abundance between oocytes and embryos. Only loci with significant differential transcript abundance based on contrasts between contiguous stages are represented (i.e.: MII oocyte versus GV oocyte, eight-cell embryos versus MII oocyte, blastocysts versus eight-cell embryos). Figure 3A was created with BioRender

In order to understand the pattern of regulation of the novel and potential novel genes during pre-implantation development, we focused our analysis of differential transcript abundance on the 1999 loci that are novel and annotated (1705) or potential novel genes (294) (Fig. 3, Additional files 4–9). Most (92%) of the loci showed alteration in transcript abundance between two developmental stages. Not surprisingly, comparisons between oocytes and embryos (eight-cell or blastocyst) resulted in a greater number of loci with differential transcript abundance (Fig. 3A). On the other hand, it was interesting to observe that 312 loci had differential transcript abundance between eight-cell and blastocyst stages (Fig. 3A).

Changes in transcript abundance during the pre-implantation development revealed that several genes followed four distinct patterns of expression (Fig. 3B). The most common pattern (717 loci) was the depletion (FDR < 0.01) of transcripts between oocytes and eight-cell stage followed by a non-significant change in transcript abundance between eight-cell and blastocyst stage. Next, 488 loci showed an increase (FDR < 0.01) in transcript abundance between oocytes and eight-cell stage followed by a non-significant change in transcript abundance between eight-cell and blastocyst stage. The other two patterns were the continuous depletion or increase of transcript abundance between oocytes, eight-cell and blastocyst stage (182 and 34 loci respectively, FDR < 0.01).

### Functional characterization of novel and putative novel loci in pre-implantation embryos

To better understand the function of novel annotated genes and potential new loci in pre-implantation embryo development, we conducted a co-expression analysis between 552 loci that showed an increased transcript abundance in embryos relative to MII oocytes (3rd and 4th patterns in Fig. 3A) and the 12,932 protein-coding and long non-coding genes that have been annotated. We also focused the analysis on eight-cell and blastocyst embryos.

**Fig. 4:**
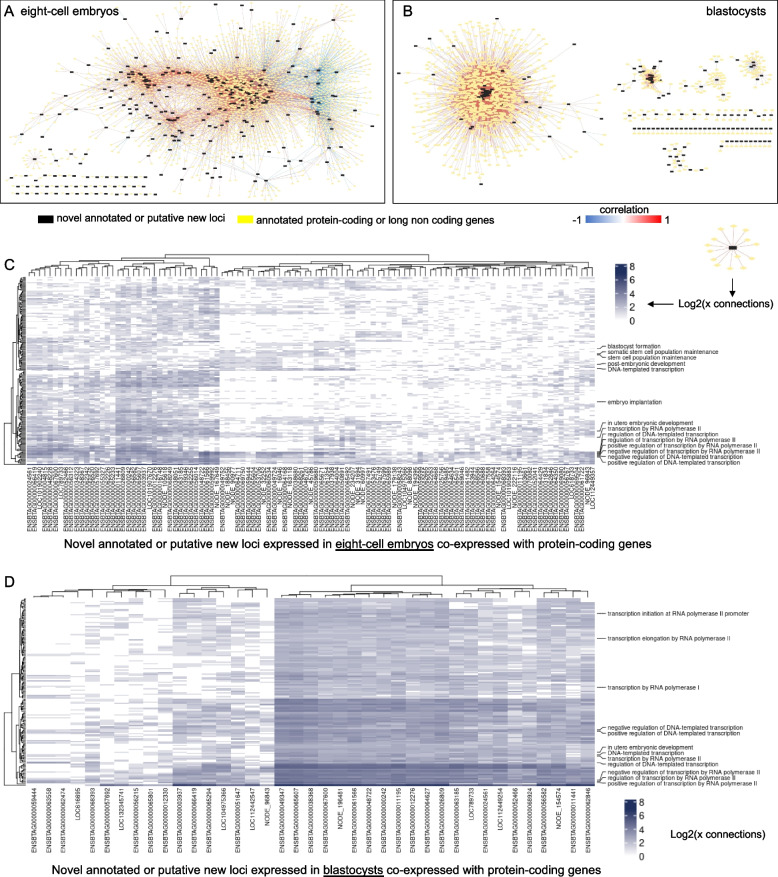
In silico functional characterization of the novel annotated genes and putative new loci. Co-expression networks with annotated protein-coding genes and long non-coding genes for eight-cell stage (**A**) and blastocysts (**B**). Heatmaps of connectivity for eight-cell embryos (**C**) and blastocysts (**D**) based on the co-expression networks with genes that are functionally annotated in gene ontology database

In eight-cell embryos, there were 247 novel annotated genes and 41 putative new loci showing significant co-expression with 2761 annotated genes (|r|> 0.85, P ≤ 1 × 10^–8^, Fig. 4A, Additional file 10). Interestingly, biological processes “translation” (167 genes), “ribosomal small subunit biogenesis” (33 genes) and “rRNA processing” (42 genes) were enriched among the 2761 co-expressed genes (FDR < 0.01, Additional file 12). In blastocysts, there were 157 novel annotated genes and 23 putative new loci showing significant co-expression with 1667 annotated genes (|r|> 0.85, P ≤ 5 × 10^–7^, Fig. 4B, Additional file 11). Those 1667 showed enrichment for “translation” (154 genes), “ribosomal large subunit biogenesis” (24 genes), “ribosome biogenesis” (29 genes), “rRNA processing” (33 genes), “ribosomal small subunit biogenesis” (27 genes) and “RNA splicing” (36 genes) biological processes (FDR < 0.01, Additional file 13).

A careful interrogation of the genes co-expressed with novel or putative new loci also revealed that many novel genes were highly correlated with several genes involved in the regulation of transcription in both eight-cell and blastocyst stages (Fig. 4C-D). Most notably, at eight-cell stage, 22 putative new genes and 80 novel annotated genes showed co-expression with genes (*BRAF*, *BYSL*, *EIF4ENIF1*, *ERRFI1*, *FOXO3*, *FUT10*, *GABPA*, *GNL3*, *IGF2BP1*, *KLF4*, *KLF10*, *LEO1*, *MYC*, *NANOG*, *NFIB*, *PAF1*, *PCM1, SUPT6H*, *PRDM14*, *PROX1*, *RIF1*, *RBPJ*, *RPL7L1*, *RRP7*, *SIRT6*, *SF3B6*, *SOX4I, STAT3*, *TBX3*, *TEAD4*, *TPT1*, *TRIM28*, *VMP1*, *WDR74*, *WDR43*, *ZP3*, and *ZHX2*) that are annotated with biological processes extremely relevant for embryo development (“blastocyst formation”, “stem cell population maintenance” and “embryo implantation”, Fig. 4C). These co-expressing pairs of genes were not present in blastocysts (Fig. 4D).

**Fig. 5:**
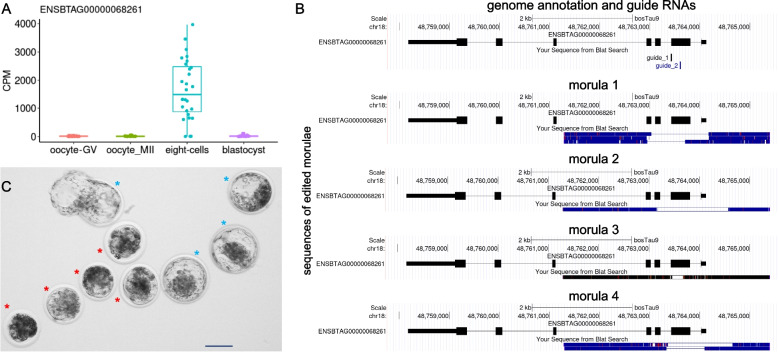
Gene editing to evaluate the importance of novel genes in pre-implantation development. **A** Transcript abundance of the novel gene ENSBTAG00000068261 in individual oocytes, eight-cell embryos and blastocysts. **B** Examples of sequences containing deletions of exon 2 of the ENSBTAG00000068261 novel gene. **C** Representative images of the embryos that arrested development at the morula stage (red asterisk) compared to control blastocysts (blue asterisk, all collected and imaged ~ 190hpf). Scale bar represents 100 µm

We selected the novel gene ENSBTAG00000068261 for further validation via CRISPR-Cas9D10A approach. This gene was selected because of its abundant transcription in eight-cell embryos (Fig. 5A) and co-expression with genes associated with “cell differentiation” (*ALKBH1*), “blastocyst formation” (*SUPT6H*), “regulation of blastocyst development” (*KLF4*), “regulation of transcription by RNA polymerase II” (*KLF4*, *KLF17*, *SNAI1*, *ZNF394*, *ZNF570*, *ZNF608*, *ZIM3*, *ZSCAN4*), and “stem cell population maintenance” (*KLF4*) (Additional files 10 and 12). The introduction of ribonucleoproteins targeting exon 2 of ENSBTAG00000068261 into zygotes caused deletions in the targeted sequence (Fig. 5B). Embryos subjected to editing displayed similar cleavage rates when compared to controls (ribonucleoprotein with scrambled guide RNA), accessed at ~ 45 h post fertilization (hpf, 70.5% ± 12.7 versus 74.2% ± 6.09; *P* = 0.531). By contrast, there was a significant reduction in blastocysts developed relative to controls at ~ 168hpf (7.52% ± 4.57 versus 19.8% ± 4.57; *P* = 1.57 × 10^–3^) and ~ 190hpf (11.4% ± 6.32 vs. 36.3% ± 6.85; *P* = 1.55 × 10^–7^)(Additional files 14–15). A greater proportion of embryos arrested development at the morula stage (Fig. 5C).

## Discussion

Our driving motivation was to determine whether there are genes that have yet to be identified in the cattle genome. Here, we carried out a hybrid transcriptome reconstruction using high-throughput sequencing data collected from oocytes and early developing cattle embryos. We report 3,033 potential new genes with no annotation in major genomic databases (Ensembl [48], NCBI/RefSeq [56] or NONCODEv5 [57]) along with 9,052 loci recently (January/2024) added to Ensembl and RefSeq annotations. It was notable that a set of newly identified genes can integrate gene regulatory networks with genes involved in biological functions essential for embryo development. We also demonstrated the importance of those new genes by disturbing its expression in pre-implantation embryos, which led to developmental arrest at the morula stage. The results confirm our hypothesis and reveal an important gap in our understanding of the genome function in the early stages of development.

Our research aimed to identify new genes in the cattle genome expressed in oocytes and pre-implantation embryos. Our analysis focused exclusively on in vitro-produced embryos, which reliably recapitulate in vivo pre-implantation development with great, but there are known differences in the expression of in vivo versus in vitro-produced embryos [13, 86]. Second, we did not generate ONT long-reads from eight-cell embryos. However, this limitation did not impact the transcriptome reconstruction, as rnaSPAdes relies on short-reads to build contigs and only utilizes long-read sequences to close gaps. Our findings increase our ability to understand pre-implantation embryo development and identify genes involved in early embryonic arrest.

The identification of 3,033 potential new genes may seem high, however, congruent lines of evidence indicate that the results are not artifacts. First, the use of a hybrid data, combining short and long-reads, is beneficial for identifying new loci [29] and produces contigs in de novo assemblies with high confidence [87, 88]. The number of contigs that we report were also filtered by those that contained a minimum of short reads mapping to them, thus increasing our confidence of detection. Second, in the process of concluding our study, a new annotation was released in January/2024, and 2238 transcripts or contigs that were initially categorized as potential new genes were reassigned to novel genes (see Table 2). Thus, the most recent annotation from Ensembl or NCBI corroborated 42% of our initial loci (2238/5271). Third, beyond bioinformatic predictions conducted by NCBI and Ensembl, the samples we used in our study (oocytes and blastocysts) are not commonly included in similar research previously executed [29, 89, 90]. Even in our samples, the putative new genes were not expressed at high levels. Fourth, the pattern of expression of those potential new genes, discussed further below, is indicative of functional units in the genome [91, 92]. Thus, the findings indicate that the new transcripts identified in our de novo transcriptome assembly are from putative new genes.

Since there was limited information related to the recently identified new genes and no data related to potential new genes, we performed a classification step and identified that most of the gene loci were associated with at least one TE by coordinate overlapping, which is coherent with previous observations in mice [93]. Such a high number of associations could be explained, first, by the high TE activity and expression during germline and pluripotent-like cells, such as oocytes and embryos in mammals [94, 95], which are suggested to play important roles in pluripotency maintenance [94] and transcriptional regulation [96, 97]. Second, TE are not randomly distributed in the genome, and it has been co-located with more active genes along with developmental stages [97, 98]. Notwithstanding, TE have been involved in the origin of new lncRNA [99, 100], and have been identified in several protein-coding sequences [101–103], which supports our findings.

Several of the novel genes and possible new genes had significant differential transcript abundance in eight-cell embryos relative to oocytes. This finding is similar to patterns previously identified in the literature considering that a major embryo genome activation occurs in cattle at the 8-cell stage [11, 104, 105]. The transcriptional activation of novel genes and possible new genes at the eight-cell stage supports the fact that those loci are actual genes and the rules of their regulation follow the annotated protein-coding and long non-coding genes.

The increase in transcripts for several genes at the eight-cell stage is a strong indication of their importance for major events that follow the embryo genome activation. To that end, the fact that most of the annotated co-expressing genes were related to translation, indicates that hundreds of those uncharacterized genes produce proteins that either have a direct role in protein synthesis or participate in the regulation of downstream genes directly related to protein synthesis. The notion that those uncharacterized genes participate in the regulation of other genes is also supported by the co-expression of hundreds of uncharacterized genes with genes annotated with transcription related to biological functions. Another in silico layer of support for the importance of those novel genes and possible new genes (522 genes) was their co-expression with several genes annotated with biological functions related to embryo development and/or stem cell pluripotency, such as *KLF4* [106–108]*, ALKBH1* [109, 110]*,* and *SUPT6H* [111, 112]. Collectively, the increase in transcription at the eight-cell stage and co-expression with genes that are important for embryo development support their critical role in the early stages of development.

The different expression patterns of novel genes and potential new genes during early embryonic development stages indicate a stage-specific importance that could also be associated with TE. Repetitive elements have a dynamic regulation of TEs in human [21] and mouse [20] pre-implantation embryos and some of those families are suggested to be expressed pluripotency-stage-specifically [113, 114]. Such a relationship is also suggested due to the presence of the same transcription factor binding sites located by TE [115, 116], which explains the high correlation with genes enriched in transcriptional and translation regulation, and more specifically, stem cells population maintenance and embryo/blastocyst development, such as *STAT3, PRDM14*, and *NANOG*, which are well-known to be involved in pluripotency and embryo development in cattle [117–120].

In order to test how critical those novel or potential new genes are for embryo survival, we deleted exon 2 of the gene ENSBTAG00000068261, also identified as LOC132342749 on RefSeq/NCBI database. This gene was originally selected for gene editing as a potential new gene but was annotated as a novel gene in January/2024, receiving the description of “F-box only protein 27-like”. As a member of the F-box protein genes, it is possible that F-box only protein 27-like participates in the SFC complex, which is comprised of Cullin, RBX1, and SKP1 proteins, all of which have an important role in the ubiquitination of maternal proteins during the maternal-to-zygotic transition [121]. The SFC complex also participates in the regulation of cell proliferation and differentiation [122, 123], which is aligned with the co-expression between transcripts of F-box only protein 27-like and other genes known to regulate pluripotency. A role of F-box only protein 27-like in pluripotency is also supported by embryonic arrest at the morula stage when loss-of-function in induced in zygotes.. A Similar phenotype was observed by Kinterova et al. [124] when SFC complex activity was inhibited. The findings are an example that several genes that remain poorly studied have important biological implications in early embryo development.

## Conclusion

Fifteen years after the release of the first draft of the cattle genome [125], our study reveal that thousands of genes have yet to be annotated. The findings also provide multiple lines of evidence that possible new genes have a critical role in early stages of embryo development, some of which are necessary for the correct formation of blastocysts. The findings herein add important insights to the complex biology involving genes expressed in oocytes and embryos and their role in the acquisition of developmental competence to achieve a successful pregnancy.

### Supplementary Information

Additional file 1: Table S1. Table with oligonucleotide sequences. Table with oligonucleotide sequences used in this study. 

Additional file 2: Table S2. Pre-processing RNA-seq short-reads samples summary. Number of paired-end reads per sample and the total number of pre-processed reads used in de novo assembly.

Additional file 3: Table S3. Hybrid de novo transcriptome assembly. Summary of sequences generated by RNAspades using pre-processed Illumina PE short-reads and super-accuracy basecalled ONT long-reads. Summary of DESeq2 and EdgeR output tables for differentially expressed potential gene loci and reference novel gene loci.

Additional file 4: Table S4. Summary of differentially expressed genes (Oocyte - Germinal Vesicle vs. Oocyte - Metaphase II). Summary of DESeq2 and EdgeR output tables for differentially expressed potential gene loci and reference novel gene loci.

Additional file 5: Table S5. Summary of differentially expressed genes (Oocyte - Germinal Vesicle vs. Eight-cell). Summary of DESeq2 and EdgeR output tables for differentially expressed potential gene loci and reference novel gene loci.

Additional file 6: Table S6. Summary of differentially expressed genes (Oocyte - Germinal Vesicle vs. Blastocyst). Summary of output tables of DESeq2 and EdgeR for differentially expressed potential novel gene loci and reference novel gene loci.

Additional file 7: Table S7. Summary of differentially expressed genes (Oocyte - Metaphase II vs. Eight-cell). Summary of DESeq2 and EdgeR output tables for differentially expressed potential gene loci and reference novel gene loci.

Additional file 8: Table S8. Summary of differentially expressed genes (Oocyte - Metaphase II vs. Blastocyst). Summary of DESeq2 and EdgeR output tables for differentially expressed potential gene loci and reference novel gene loci.

Addditional file 9: Table S9. Summary of differentially expressed genes (Eight-cell vs. Blastocyst). Summary of DESeq2 and EdgeR output tables for differentially expressed potential gene loci and reference novel gene loci.

Additional file 10: Table S10. Summary of co-expression (Eight-cell). Table retrieved from the co-expression matrix of DE novel genes/potential novel genes with annotated genes (|r| ≥0.85 and *p*-value ≤ 0.05) in eight-cell samples that followed pattern #3 and #4 (Fig. 3).

Additional file 11: Table S11. Summary of co-expression (Blastocyst). Table retrieved from the co-expression matrix of DE novel genes/potential novel genes with annotated genes (|r| ≥0.85 and *p*-value ≤ 0.05) in blastocyst samples that followed pattern #3 and #4 (Fig. 3).

Additional file 12: Table S12. Summary of the functional enrichment (Eight-cell). Table retrieved from functional enrichment of co-expressed annotated genes (|r| ≥0.85 and *p*-value ≤ 0.05) with DE genes in Eight-cell samples that followed the pattern #3 and #4 (Fig. 3).

Additional file 13: Table S13. Summary of the functional enrichment (Blastocyst). Table retrieved from functional enrichment of co-expressed annotated genes (|r| ≥0.85 and *p*-value ≤ 0.05) with DE genes in blastocyst samples that followed the pattern #3 and #4 (Fig. 3).

Additional file 14: Table S14. Embryo developmental rates for CRISPR-Cas9D10 targeting ENSBTAG00000068261 in scramble controls and genome-edited samples. Description of data: Embryo developmental rates for CRISPR-Cas9D10 targeting ENSBTAG00000068261 in scramble controls and genome-edited samples. hpf: hours post fertilization; %: percentage of embryos relative putative zygotes; SD: standard deviation.

Additional file 15: Table S15. Contrasts between scramble controls and genome-edited groups for blastocyst yield at 168hpf. Contrasts between scramble controls and genome-edited groups for blastocyst yield at 168hpf. SE: standard error; DF: degrees of freedom; adj p-value: Bonferroni corrected *p*-values.

## Data Availability

All raw and processed sequencing data generated in this study have been submitted to the NCBI Gene Expression Omnibus (GEO; https://www.ncbi.nlm.nih.gov/geo/) under accession identifiers GSE99678, GSE199210, GSE225693.

## Abbreviations

8c: Eight-cell
*ALKBH1*: AlkB homolog 1, histone H2A dioxygenase
BL: Blastocyst
BP: Biological process
*BRAF*: B-raf proto-oncogene, serine/threonine kinase
*BYSL*: Bystin like
cDNA: Complementary DNA
COCs: Cumulus-oocyte complexes
CPM: Counts per million
DE: Differentially expressed
*EIF4ENIF1*: Eukaryotic translation initiation factor 4E nuclear import factor 1
*ERRFI1*: ERBB receptor feedback inhibitor 1
FDR: False discovery rate
*FOXO3*: Forkhead Box O3
*FUT10*: Fucosyltransferase 10
*GABPA*: GA binding protein transcription factor subunit alpha
*GNL3*: G Protein nucleolar 3
gRNA: Guide RNA
GV: Germinal vesicle
HEPES: 4-(2-Hydroxyethyl)-1- piperazineethanesulfonic acid
HEPES-SOF: HEPES buffered synthetic oviductal fluid
hpf: Hours post-fertilization
*IGF2BP1*: Insulin like growth factor 2 MRNA binding protein 1
IVC: in vitro culture
IVF: in vitro fertilization
IVM: in vitro maturation
*KLF4*: Krueppel-like factor 4
*KLF10*: Krueppel-like factor 10
*KLF17*: Krueppel-like factor 17
lncRNA: Long noncoding RNA
*LEO1*: LEO1 homolog, Paf1/RNA polymerase II complex component
LR: Long-read
MII: Metaphase II
mRNA: Messenger RNA
*MYC*: Myc proto-oncogene
*NANOG*: Nanog homeobox
*NFIB*: Nuclear factor I B
ONT: Oxford nanopore technologies
ORFs: Open read frames
*PAF1*: PAF1 homolog, Paf1/RNA polymerase II complex component
PBS: Phosphate buffered saline solution
*PCM1*: Pericentriolar material 1
*PRDM14*: PR/SET domain 14
*PROX1*: Prospero homeobox 1
PZ: Putative zygote
*RBPJ*: Recombination signal binding protein for immunoglobulin kappa J region
*RBX1*: Ring-box 1
*RIF1*: Replication timing regulatory factor 1
RNPs: Ribonucleoproteins
*RPL7L1*: Ribosomal protein L7 Like 1
*RRP7*: Ribosomal RNA processing 7
*SF3B6*: Splicing fFactor 3b subunit 6
SFC: Skp, Cullin, F-box containing complex
sgRNA: Single guide RNA
*SIRT6*: Sirtuin 6
*SKP1*: S-phase kinase associated protein 1
*SNAI1*: Snail family transcriptional repressor 1
SOF-BE: Synthetic oviductal fluid (SOF) fertilization medium bovine embryo
SOF-Fert: Synthetic oviductal fluid (SOF) fertilization medium
*SOX4I*: SRY-box transcription factor 4I
SR: Short-reads
*STAT3*: Signal transducer and activator of transcription 3
*SUPT6H*: SPT6 homolog, histone chaperone and transcription elongation factor
*TBX3*: T-box transcription factor 3
TE: Transposable element
*TEAD4*: TEA domain transcription factor 4
*TPT1*: Tumor protein, translationally-controlled 1
*TRIM28*: Tripartite motif containing 28
UCSC: University of California Santa Cruz
UTRs: Untranslated regions
*VMP1*: Vacuole membrane protein 1
*WDR43*: WD repeat domain 43
*WDR74*: WD repeat domain 47
*ZHX2*: Zinc fingers and homeoboxes 2
*ZIM3*: Zinc finger imprinted 3
*ZNF394*: Zinc finger protein 394
*ZNF570*: Zinc finger protein 570
*ZNF608*: Zinc finger protein 608
*ZP3*: Zona pellucida glycoprotein 3
*ZSCAN4*: Zinc finger and SCAN domain containing 4
χ2: Chi-squared
